# STEM: An Open Source Tool for Disease Modeling

**DOI:** 10.1089/hs.2019.0018

**Published:** 2019-08-19

**Authors:** Judith V. Douglas, Simone Bianco, Stefan Edlund, Tekla Engelhardt, Matthias Filter, Taras Günther, Kun (Maggie) Hu, Emily J. Nixon, Nereyda L. Sevilla, Ahmad Swaid, James H. Kaufman

**Affiliations:** Judith V. Douglas, MHS, was Lead Technical Writer, Science to Solutions; Simone Bianco, PhD, is a Research Staff Member, Industrial and Applied Genomics, Science to Solutions; Stefan Edlund, MS, is a Research Software Engineer, Industrial and Applied Genomics, Science to Solutions; Kun (Maggie) Hu, PhD, is Research Manager, Public Health and Food Safety; and James H. Kaufman, PhD, is Chief Scientist, Science to Solutions; all at IBM Research—Almaden, San Jose, CA.; Dr. Bianco is also with the National Science Foundation Center for Cellular Construction, University of California San Francisco.; Tekla Engelhardt, PhD, is an Analyst, System Management and Supervision Directorate, National Food Chain Safety Office, Budapest, Hungary.; Matthias Filter, Dipl, is a Research Scientist; Taras Günther, MSc, is a PhD student and Scientific Assistant; and Ahmad Swaid is a Software Developer; all in the Biological Safety Department, Food Hygiene and Technology, Supply Chains and Food Defense, German Federal Institute for Risk Assessment, Berlin, Germany.; Emily J. Nixon is a PhD student, School of Biological Sciences, University of Bristol, UK.; Nereyda L. Sevilla, PhD, is an Aerospace Physiologist, Schar School of Policy and Government, George Mason University, Fairfax, VA.

**Keywords:** Epidemic management/response, Infectious diseases, Viral hemorrhagic fevers, Influenza, Open source epidemiologic modeling

## Abstract

The Spatiotemporal Epidemiologic Modeler (STEM) is an open source software project supported by the Eclipse Foundation and used by a global community of researchers and public health officials working to track and, when possible, control outbreaks of infectious disease in human and animal populations. STEM is not a model or a tool designed for a specific disease; it is a flexible, modular framework supporting exchange and integration of community models, reusable plug-in components, and denominator data, available to researchers worldwide at www.eclipse.org/stem. A review of multiple projects illustrates its capabilities. STEM has been used to study variations in transmission of seasonal influenza in Israel by strains; evaluate social distancing measures taken to curb the H1N1 epidemic in Mexico City; study measles outbreaks in part of London and inform local policy on immunization; and gain insights into H7N9 avian influenza transmission in China. A multistrain dengue fever model explored the roles of the mosquito vector, cross-strain immunity, and antibody response in the frequency of dengue outbreaks. STEM has also been used to study the impact of variations in climate on malaria incidence. During the Ebola epidemic, a weekly conference call supported the global modeling community; subsequent work modeled the impact of behavioral change and tested disease reintroduction via animal reservoirs. Work in Germany tracked salmonella in pork from farm to fork; and a recent doctoral dissertation used the air travel feature to compare the potential threats posed by weaponizing infectious diseases. Current projects include work in Great Britain to evaluate control strategies for parasitic disease in sheep, and in Germany and Hungary, to validate the model and inform policy decisions for African swine fever. STEM Version 4.0.0, released in early 2019, includes tools used in these projects and updates technical aspects of the framework to ease its use and re-use.

## Abstract 2

The Spatiotemporal Epidemiologic Modeler (STEM) is an open source software project supported by the Eclipse Foundation and used by a global community of researchers and public health officials working to track and, when possible, control outbreaks of infectious disease in human and animal populations. STEM is not a model or a tool designed for a specific disease; it is a flexible, modular framework supporting exchange and integration of community models, reusable plug‐in components, and denominator data, available to researchers worldwide.

The Spatiotemporal Epidemiologic Modeler (STEM) is an open source software project used by a global community of researchers and public health officials working to track and, when possible, control outbreaks of infectious disease in human and animal populations.^1^ STEM is not a model or a tool designed for a specific disease; it is a flexible, modular framework supporting exchange and integration of community models and data. The US Air Force/Surgeon General provided early sponsorship for the development of STEM; today, IBM Research and the German Federal Institute for Risk Assessment (BfR) play leadership roles in the STEM community.

STEM differs from other open source projects^2,3^ in that it has a graphic user interface (GUI) and runs on Eclipse Equinox,^4^ an industry standard framework offered and supported by the Eclipse Foundation. In this framework, every model component is an independent building block, or “plug-in,” available for reuse in the GUI through drop and drag. The software components can be shared back to Eclipse for review and approval as open source contributions, or they can be kept proprietary (and private) under Eclipse's flexible non-viral license.^5^

Using the STEM website, researchers can apply, adapt, or extend specific models and classes of models available as free and open resources.^6,7^ STEM also provides many gigabytes of public denominator data from sources including the United Nations, the World Health Organization (WHO), the US Census, the National Oceanic and Atmospheric Administration, and DIVA-GIS for use with any model.^8^

The capabilities that STEM and the Eclipse framework provide are perhaps best illustrated by the range of work done by members of the STEM community, as summarized in Table 1 and described in this article.

**Table 1.** An Overview of Projects Using STEM

*Disease*	*Affiliations*	*Research Question*	*Type of Model*	*Conclusions*	*Contributions/Value*
Seasonal influenza in Israel	Israel Center for Disease Control; Maccabi Healthcare Services, Israel; IBM Haifa: Israel Ministry of Health; IBM Research, USA	Are there differences between the seasonal transmission of influenza A and B?	Three deterministic SIRS models to compare serotypes: (1) control [identical models], (2) effect of differing transmission rates, (3) effect of differing transmission rates, seasonal forcing, and flu season length	• Influenza A was dominant in 8 of 10 years, B in 2 years • Second model improved predictability for both influenza A and B with excellent fit with historic data • Third model was shown to be overdetermined	• Gathered/analyzed 10 years of surveillance data from Israeli CDC and patient data from Maccabi Healthcare • Contributed 3 models to STEM for re-use • Provided the Israeli CDC insight into vaccine development for seasonal influenza
H1N1 in Mexico City	Mexico City Government; IBM Research, USA	Were social distancing measures implemented in response to the epidemic in the city effective?	SIRS model used in the Israeli study and extended to assess changes in the transmission rate during intervention; automated simulation optimization used to discover intervention date and duration	• Simulations showed 22% reduction in transmission rate during the period schools were closed • Results validated, using confirmed incidence data from the epidemic	• Demonstrated effectiveness of social distancing measures that had been controversial at the time they were implemented • Framework for STEM interventions extended to support optimization against data
Measles in London	National Institute for Health Research and National Health Service, United Kingdom; Northwestern University, USA; IBM Research	What are the likely outcomes of 2 local vaccination policy changes (target all clinics or a subset of poor performers)?	SEIR model extended to include aging population demographics	• In 5 years, targeting 10% increase in coverage in all 73 clinics would result in 26 cases/year • Targeting only the 8 poorest performing clinics would result in 34 cases/year	• Demonstrated ability to model control measures, such as vaccinations, in simulations • Added interventions framework • Provided results to policymakers for cost/benefit analysis • Demonstrated use of modeling to inform resource allocation
H7N9 avian influenza in China	Chinese Center for Disease Control; IBM Research	Assuming no effective public health intervention, what is the epidemic size for the human population (1) if the transmission remains only from birds-to-humans, and (2) if the virus evolves to transmit human-to-human?	Susceptible Infectious Recovered (SIR) models for (1) human host and (2) bird-host transmission to estimate epidemic size for transmission only from bird-to-human or for evolved human-to-human	• Used wild bird migration pattern (Martcheva) and daytime temperature data available in STEM to run stochastic and deterministic simulations	• Validated stochastic solvers using a random pick from a binomial distribution • Revealed a bug in the Apache Commons Mathematics Library, fixed in Apache math V3.0 • Using limited information, created risk maps for Chinese CDC
Dengue in Thailand	University of California San Francisco and IBM Research, USA	In cases of dengue, a vector-borne disease with 4 strains, what factors confer immunity to or increase risk from re-infection?	Three deterministic models of increasing complexity: (1) host only, (2) host plus mosquito vector, and (3) mosquito vector plus host incubation period	• Results in all 3 models showed cross immunity alone did not explain periodic outbreaks; levels of antibody dependent enhancement were also involved • First model showed highly chaotic behavior; the 2 more complex models diminished instability	• Further work is needed to clarify the role of seasonality, demographics, environmental and climate changes • Including the mosquito vector may help calibrate models based on surveillance data and achieve greater predictability
Ebola in West Africa	IBM Research and global community listed at https://wiki.eclipse.org/Ebola_Call_Participants	What interventions based on human behavioral changes could help contain the Ebola outbreak in West Africa?	Susceptible Exposed Infectious Recovered (SEIR) model extended to capture 4 transmission pathways	Two interventions together reduced the reproduction number below 1.0: (1) isolation or hospitalization of infectious patients within 2.5 days of onset of symptoms, and (2) burial of infectious corpses within 34 hours	• Evaluated impact of human behavior change as an intervention • Recognized by the White House Office of Science and Technology Policy
	Montclair State University, NJ, USA; IBM Research	What effect does the animal reservoir for Ebola have on its potential reintroduction in humans?	Deterministic and stochastic analysis of disease and population parameters	Reservoir has important role in preventing disease extinction	In presence of an active reservoir, asymmetric human birth and death rates (1) increase the potential of endemic disease in relatively small population while they (2) prevent large outbreaks
Malaria in Thailand	King Mongkut's University of Technology, Thailand; Johns Hopkins School of Public Health; and IBM Research, USA	How do fluctuations in climate variables affect global malaria incidence?	Macdonald Ross vector model/ climate-driven vector capacity model	• Correctly predicted relative malaria change in ∼75% of endemic countries reported by WHO • Suggested greater predictability possible with higher spatial resolution	• Demonstrated ability to incorporate earth science data in STEM models • Accessed climate data from the National Oceanic and Atmospheric Administration and made it available as plug-ins for re-use in other research projects
Salmonella in Germany	Federal Institute for Risk Assessment (BfR), Germany	Is it possible to model the spread and transmission of food-borne diseases from farm to fork?	Susceptible Infectious Recovered (SIR) model for pigs and humans; susceptible infectious (SI) model for pork Extended to include transport and population transformation models	Demonstrated the STEM framework can handle complex supply chain models including time- and location-specific transportation events as well as the transformation of entities via food production and processing events	Contributed new features to STEM • Shapefile Graph Generator for proprietary geo-coded data files • Pajek Net Graph Generator for transportation events • Graph Editor for visualization and node/edge properties • Transformation Decorator
SARS, H1N1, Ebola and pneumonic plague in air travel	George Mason University, USA	What is the role of air travel as a vector in infectious disease transmission?	• Deterministic Susceptible Exposed Infectious Recovered (SEIR) model to control for environmental and population data using air transportation routes while exploring characteristics of the diseases	• SARS and H1N1 pose an air travel threat that is validated with historic data • Ebola is not spread by aerosol but by bodily fluids and does not pose an air travel threat, also validated by historical accounts • Pneumonic plague, though spread by aerosol, does not pose an air travel threat due to rapid illness and death	• Demonstrated air travel model revealing the role an aircraft may have as a vector and as an incubator for the spread of infectious diseases • Clarified disease characteristics of concern in assessing and recommending biosecurity countermeasures during air travel
Parasitic livestock disease in the United Kingdom	University of Bristol, United Kingdom	What are the most effective strategies to control transmission of livestock parasitic disease?	• Within and between farm transmission model	Work ongoing	• Demonstrates use of STEM's new stochastic solver, large pajek graph feature, and model creator • Offers approach to look at ways of improving control strategies for parasitic disease in livestock
African swine fever in Germany and Hungary	Federal Institute for Risk Assessment (BfR), Germany	Is it possible to re-implement, validate, and extend the African swine fever model developed by Barongo et al^73^?	• Re-implemented Barongo's base and bio-intervention models • Extended using a more complex population model to include mixing rates between different subgroups of the wild boar population • Inclusion of mixing rates had a huge impact on the simulated spread of the disease	• Results of 1,000 simulations for the base model were consistent with Barongo's; those for the bio-intervention models were not.	• Demonstrated approach to re-implement parameterized epidemiologic models from literature • Potential use case in intervention planning, eg, targeted hunting or fencing of wild boars in outbreak areas
	National Food Chain Safety Office, Hungary	What enforcement actions to fight the spread of African swine fever should policymakers evaluate?	Modified Barongo's model using geospatial data and estimates of wild boar density from National Game Management Database	Identified 2 areas at risk: 1 from contaminated meat or food waste brought in by non-EU workers; the other from natural spread via migratory wild boars	• Developed evidence-based policy recommendations • Work is ongoing

*Note.* All are compartment models: Susceptible Infectious (SI), Susceptible Infectious Recovered (SIR), Susceptible Infectious Recovered Susceptible (SIRS), Susceptible Exposed Infectious (SEI), Susceptible Exposed Infectious Recovered (SEIR).

## STEM Projects

### Seasonal Influenza in Israel: Variations in Transmission by Strains

Some of the most widely studied epidemiologic models are for influenza, both pandemic and seasonal. Because of the fleeting herd immunity characteristic of flu, such models typically use a deterministic compartmental disease model, in which individuals move from susceptible, to infectious, to recovered, and once again become susceptible (SIRS). For seasonal flu, which appears in the northern hemisphere every winter and virtually disappears during the summer months, the subtlety involved in modeling relates to the way seasonality is introduced, and how that seasonality may vary for different strains of the virus.^9-11^ Predictions of upcoming flu seasons are critical to vaccine development but remain imprecise.

Working with Israeli researchers, members of the STEM community developed and ran 3 models using 10 years of incidence data for seasonal flu collected by the Israeli Center for Disease Control from health clinics across the country.^12,13^ In 8 years, strain *A* was more frequently identified; in 2 years, strain *B*. The first model (a control) assumed no differences between the 2 strains; the second allowed for different transmission rates; and the third expanded to include different background transmission and flu season length. Using STEM's automated experiment (Nelder-Mead) plug-in,^14^ the project team cross-validated the models by fitting them to the historic data for a subset of the 10 years and then attempting to predict the incidence for the remaining years. Results showed the second model, which accounted for variations in transmission, increased the predictive ability for both strains, while the third model did not.

The team concluded that the next step to improve predictive capabilities would be to fit a full multistrain model of influenza, but they questioned whether the available data were adequate to do so.^13^

### H1N1 in Mexico City: Evaluation of Social Distancing as Prevention

**Figure 1.**
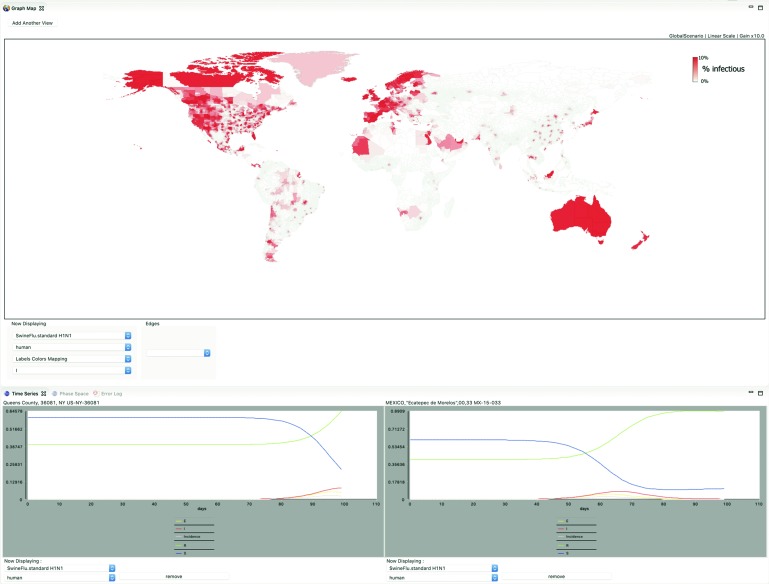
A Global H1N1 Pandemic Figure 1 is a screen shot of a global H1N1 simulation with air travel (running on a MacBook Pro™). The map is set to display the “infectious” population with a color scale gain x10, so the red color saturates at or above 10% infectious as indicated by the color bar in the map view. The 2 time series charts at the bottom of the image show the variables S, I, R, and incidence for both Ecatepec de Morelos (Mexico) and Queens County (New York). The epidemic peaked in Mexico City in slightly under 60 days from simulation start (with a single patient zero). The screen shot was taken just before 100 days at epidemic peak in Queens County (lower left time series). Note that this particular pandemic scenario was initialized with only 40% initial herd immunity as shown by initials *R* = 40% and *S* = 60% in both time series charts. Color images are available online.

On April 17, 2009, Mexico announced a national influenza alert, 2 days after the first H1N1 patient was confirmed by laboratory testing at the US Centers for Disease Control and Prevention. In 3 weeks, influenza accounted for 90,000 visits to 220 health units and 20 hospitals in Mexico City.^11^ The mayor's response, controversial at the time, was to implement social distancing measures, closing schools on April 24 and restaurants on April 28. All were reopened by May 11. When de-identified lab data became available, the Mexico City government collaborated with scientists at IBM Research to determine whether the actions taken had in fact limited the spread of the H1N1 virus.^15^ Using the model validated in the Israeli study, the team ran repeated global simulations, with air travel, using STEM's automated experiment feature. The transmission rate was fit based on the confirmed 2009 H1N1 incidence data for Mexico City and New York (see Figure 1). The differential equations in the SIRS model were solved using STEM's implementation of an adaptive integration algorithm,^16^ allowing the team to specify a high level of precision. Each simulation explored combinations of epidemiologic parameters, widely studied and reported elsewhere, automatically reducing the parameter step sizes until the solution approached a fit with the local minimum. The analysis determined an average reproductive number based on the values of those parameters over the 28 days between April 13 and May 10, the major epidemic wave.

To evaluate the effectiveness of social distancing, the model was extended to automatically fit a window of variable duration at any start date, where the optimizer could amplify, attenuate, or leave unchanged the base transmission rate.^15^ In searching for a start date, duration, and transmission rate scale factor that would minimize differences between the estimated and actual incidence, an arithmetic algorithm determined the window was optimally placed on April 24, the day schools were actually closed, and that the social distancing measure, controversial at the time it was implemented, was effective for a period of 6 days. Within the window, the reduction in reproductive number (and transmission rate) was ∼22%, a reduction close to that predicted earlier for the reduction in transmission expected among children.^17^

### Measles in the UK: Modeling on a Local Scale to Inform Policy

In 2012, in the wake of the anti-vaccination movement linking measles vaccine with autism, England and Wales reported more than 2,000 cases of measles, the highest figure in 2 decades.^18^ To determine whether a spatial patch model for local use could be used to inform policy change, British health researchers collaborated with members of the STEM community in a study of measles outbreaks in 2011-12 in the areas served by 73 clinics in the North West London Borough of Ealing.^19^ Immunization data were extracted for 3 cohorts, ages 1-3, 4-6, and 7-19; patient population was estimated using general practice profile records.

The project team extended the STEM model^20^ to include 2 additional compartments, 1 for maternal immunity and 1 for delays in antibody response after immunization, and generated a 20-year model of vaccination coverage for Ealing. In England, children are immunized between ages 1 and 2, then again at around age 5; hence, immunization events are modeled for the ages 1-3 and ages 4-6 cohorts. Parameter values were based on measles research literature; transmission coefficients were estimated using social contact data from the Polymod project^21^ and fitted to 2011-12 case reporting data for Ealing.

To examine possible effects of policy change, the team modeled 2 scenarios.^19^ The first increased vaccination coverage by 10% for all clinics; the second focused only on the bottom 10% of the poorest performing clinics (8 clinics total) and equivalently improved their coverage. The first scenario achieved a 58% reduction from 60 cases per year in 2011 to 26 per year in 2017; the second reduced measles by 44%, or to 34 cases per year in 2017. The project demonstrated that local scale modeling was possible and that the transparency of analysis provided by an open source application gave credence to the output of the models.

### H7N9 Avian Influenza in China: Transmission and Interventions

In 2013, amid an outbreak of H7N9 avian influenza, the Chinese CDC (CCDC) contacted the STEM modeling team at IBM to discuss how to leverage the power of the modeling approach. Their goal was to identify, prepare for, and respond to all sorts of uncertainties during the H7N9 outbreak in the absence of real-time reporting data. Collaborating with domain experts and scholars of avian influenza in China, the STEM team built a preliminary avian influenza model^22^ using the common parameter values of avian influenza to answer 2 main concerns. First, if the outbreak of H7N9 establishes sustained bird-to-human transmission, what would be the spatial diffusion pattern of H7N9 and the estimated number of epidemic size (peak infection size, total infections)? Second, if human-to-human transmission is established due to virus mutation, what would be the distribution of disease transmission, and what would be appropriate response strategies for the CCDC?

In reality, the identified cases were very sporadic at eastern and northern China during the outbreak. The team built simple compartmental SIR (susceptible-infectious-recover) models to capture the dynamics of the disease transmission between human and bird (wild and domestic) populations. The models used literature values for the avian influenza parameters published by Martcheva^23^ to initialize this H7N9 model.^24^ The bird-to-bird transmission coefficient was estimated to be about 8 times higher than human-to-human transmission of seasonal influenza. Birds, with a life expectancy of 2 years, shed viruses for 10 days on average; humans were infected for an average of 6 days and were expected to lose immunity over a period of 2 years.

To estimate the transmission coefficient between birds and humans, the parameter was calibrated so that during the course of 1 year, no more than ∼90-95 cases occurred in all of China with reported incidence.^24^ To account for seasonal variation in the transmission coefficients for bird-to-bird and bird-to-human, the model used daytime temperature data available as a plug-in in STEM. The spatial component assumed a mixing of infected birds across regions sharing a common border, with border length constraining the mixing area. A global parameter allowed the mixing to be adjusted up or down or completely turned off. When calibrating the model, the team tuned the mixing parameter to be the right value so that the first case detected in Beijing was about 50 days after the first case reported in Shanghai as observed by China CDC.

After running the first version of bird-to-human model, the STEM team modeled human-to-human transmission stochastically (more realistic) to test the hypothesis regarding possible human-to-human transmission.^24^ They experimented with a range of potential scenarios^25^ to answer what-if questions that mattered during the crisis. For example, if no effective intervention to control the disease is in place should human-to-human transmission become possible, a rough estimated total of infections in China is in the millions. Using the same model and altering particular parameter values would allow public health agencies to make quantitative evaluations of other scenarios for response planning and comparison. In an attempt to develop risk maps for the CCDC, the team ran the model deterministically and stochastically; however, the accuracy of the estimates depends on the data used to calibrate the model, such as accurate and high-resolution population density data, which were not available during the modeling practice.

### Dengue Fever: Dynamics of a Multi-Strain Vector-Borne Disease

Dengue fever is a public health threat to one-third of the world's population, and more than 400 million people are infected every year.^26^ Dengue is a vector-borne disease spread by *Aedes* mosquitoes, especially the urban species *Aedes aegypti*. There are 4 similar but distinct serotypes of dengue viruses (ie, DEN 1-4). Until recently, there was no vaccine (it was under development and trial) and no specific treatment available. In the past 50 years, dengue incidence has increased 30-fold in tropical areas,^27^ a concern in a time of climate change. The threat is compounded by the fact that immunity is life-long for only the strain responsible for the infection; cross immunity for the other 3 strains is partial and temporary.^28^ Moreover, reinfection with a different strain carries greater risk of life-threatening complications resulting from antibody-dependent enhancement (ADE) with increased risk of hemorrhagic fever.^26,29,30^

To better understand the dynamics of this complex disease, scientists at IBM Research and the University of California San Francisco used STEM to test 3 models of increasing complexity.^31^ The first “host only” model is based on work reported in the literature.^32^ The second model includes the mosquito vector, and the third adds a variable defining the incubation period in humans to the compartment model.

Results in all 3 models showed that cross immunity alone did not account for the dynamics (ie, 3-4 years outbreak periodicity) observed in the data reported from Thailand;^33^ significant levels of antibody dependent enhancement were also required. In repeated simulations, the first model showed a high degree of chaotic behavior. The other 2 more complex models diminished this instability, suggesting that explicitly including the mosquito vector may support efforts to calibrate a model based on surveillance data and to achieve greater predictability. Further work is needed to clarify the role of seasonality^31^ and other stochastic factors such as demographics and environmental and climate changes.^34^

### Ebola in West Africa: Supporting a Global Modeling Community

In 2014-15, in response to the Ebola outbreak in Africa, the STEM community convened a weekly conference call.^35^ More than 40 scientists from more than 20 research institutes, universities, government agencies, and private companies actively participated.^36^ The goal of this global community was 2-fold: to accelerate research by helping members of the scientific community interact and share data, questions, and ideas with each other; and to provide researchers critical connections with operational people responding to the outbreak. Topics ranged from strategies for early diagnosis and treatment to possible interventions, such as passenger screening at airports and behavioral measures, and to disease dynamics modeling. Notes and some deep dive presentation slides from the calls are available at the STEM website.^35^

### Ebola: Understanding the Impact of Behavioral Change

The first Ebola modeling paper published by the STEM community addressed the role of human behavioral changes in containing this highly contagious disease.^37^ Using a 7-compartment epidemiologic model to describe different Ebola transmission pathways,^38^ STEM modelers evaluated the Ebola transmission and potential impact on the affected population in West Africa using publicly available incidence report data from March 14 to August 20, 2014, from WHO. The simulation results suggested that human behavioral responses could significantly help contain Ebola transmission. Specifically, the quantitative analysis showed a significant reduction of infections when infected patients were isolated or hospitalized within 2.5 days of onset of symptoms or when infectious corpses were promptly buried within 34 hours.^37^

### Ebola: Testing Disease Reintroduction via an Animal Reservoir

Zoonotic diseases are diseases that are passed from an animal reservoir to humans, many times through intermediate hosts such as horses, monkeys, and the like. Ebola virus disease (EVD) is one such disease. While the animal reservoir for EVD has still not been identified with certainty, spillover events that originate disease outbreaks have been linked to human contacts with bats.^39^ Since the large 2014 West Africa outbreak, the dynamic of EVD has been thoroughly investigated.^38,40,41^ Little attention has been devoted to the effect of the presence of the reservoir on the reintroduction of the disease.

In collaboration with an environmental scientist and a mathematician at Montclair State University in New Jersey, IBM researchers used STEM^42^ to simulate the dynamics of EVD in the presence of the reservoir, especially with the relationship between disease and population parameters. The study has been carried out using both deterministic and stochastic methods, and the results support the important role of the disease reservoir in preventing disease extinction.^43^ In particular, the model shows how an asymmetry in human birth and death rates, together with the presence of an active disease reservoir, increase the potential for the disease to become endemic in a relatively small population, while at the same time preventing large outbreaks.

### Malaria in Thailand: Incorporating Earth Science Data in STEM Models

In collaboration with researchers from the John Hopkins Bloomberg School of Public Health, the University of Haifa in Israel, and King Mongkut's University of Technology, Thailand, a project was launched to study the effect of environmental factors on malaria incidence globally.^44^ In particular, fluctuations in climate variables such as temperature and rainfall were used as input to an *Anopheles* vector capacity model, which itself parameterized a Macdonald Ross malaria model.^45^ Using climate data from 2000 to 2010 available as a plug-in from STEM,^46^ each year was independently used to calibrate the malaria model, resulting in 10 “climate years” of malaria incidence data. By comparing all pair-wise combinations of climate years, sensitivity of malaria incidence to variations in climate variables were computed for both low- and high-resolution spatial models.^44^ In addition, the outputs of the simulated model were compared to global malaria incidence reports compiled by WHO for the years studied.

**Figure 2.**
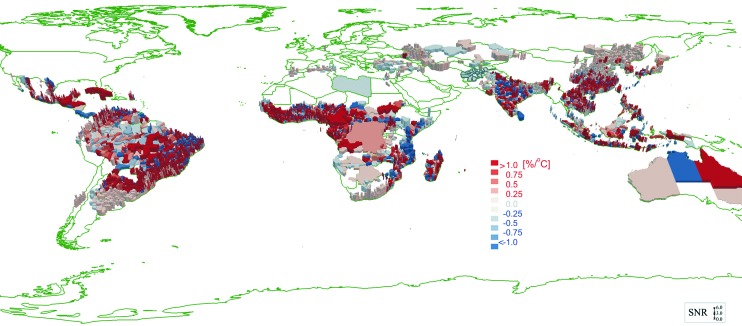
Malaria Susceptibility and Temperature Change Figure 2 shows change in malaria incidence as a function of change in temperature. The “susceptibility” (or expected response) of malaria incidence is expressed in percentage change per degree centigrade. Given that there is an optimal temperature for reproduction of the anopheles mosquito, the susceptibility can be positive or negative. Thus, in different regions, depending on average temperature, the incidence may increase (red) or decrease (blue) with increasing temperature. Color graphics available at https://www.liebertpub.com/doi/10.1089/hs.2019.0018.

The model predicted the historic year-to-year malaria burden with an accuracy of 75% for 86 countries (see Figure 2). There is significant potential for improvement in prediction accuracy if WHO were able to report incidence data at higher spatial resolution, rather than aggregating only to the national level. In addition, the model revealed which regions are most susceptible to increase malaria burden based on climate change trends.

### Salmonella in Pork in Germany: Tracking a Pathogen from Farm to Fork

In a proof-of-concept study, STEM was used to illustrate the software's capability to create models and simulations for zoonotic disease transmission scenarios.^47^ To model this specific type of disease, it was necessary to extend the classical compartment model paradigm with STEM's transformation decorator feature.^48^ This feature allows users to describe food manufacturing or food production processes (like the slaughter of animals) that cannot simply be represented by a standard compartment model. With the transformation decorator plug-in, it is possible to simulate the transformation of livestock (animals) and/or plants into raw products (meat), food items (a pizza, a salad), or animal feed. It provides the necessary means to determine how a contaminant or pathogen will be transmitted or introduced into the different production or transformation processes.

The proof-of-concept STEM model “Salmonellosis in pigs” provides an example of how to simulate the spread of a zoonotic pathogen (in this case Salmonella) from farm to fork. The example illustrates how to model the spread of Salmonella among pigs in a barn, the contamination of meat by infected carcasses in the slaughterhouses, and the infection of humans via consumption of contaminated pork meat.^49^

### Air Travel and the Spread of Diseases: Assessing Comparative Threats

Early work with STEM explored the role of air travel in the spread of diseases and developed an air traffic plug-in for STEM that included 100% of the commercial airports in the United States and 80% of those worldwide.^50,51^

**Figure 3.**
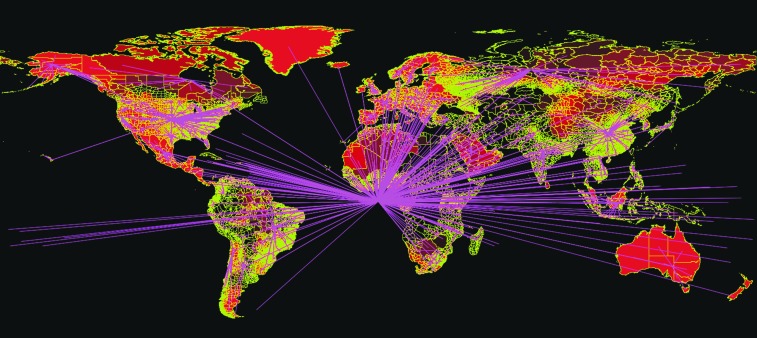
Global Air Travel Network The global air travel network available in STEM can be plugged into any disease model in STEM, as can any set of transportation edges. Air travel is modeled in analogy to fluid flow in a hierarchical network of pipes.^50^ The flow of people into or out of any location on earth is calibrated based on actual passenger travel through commercial airports worldwide. Color images are available online.

More recently, a researcher at George Mason University, in a doctoral dissertation, focused on the threat of pneumonic plague as a natural outbreak or after a bioterrorist attack.^52^ The project examined how air travel provides new means for diseases to spread internationally at unprecedented rates (see Figure 3). This was evident in the 2003 severe acute respiratory syndrome (SARS) pandemic that killed more than 800 people across 37 countries, the 2009 influenza H1N1 epidemic that affected more than 200 million individuals, and the 2014 Ebola outbreak that killed more than 11,000 people.^53-55^ An aircraft has a role in disease spread as both a vector and an incubator.^56^ An outbreak of pneumonic plague, which has a high mortality rate, is spread from person-to-person, and is endemic to the United States, may challenge the effectiveness of public health responses.^52,57–59^

To compare and contrast the spread of SARS, H1N1, and Ebola, the project extended STEM to include case fatality and infectious mortality rates based on the data of previous outbreaks, historical epidemiologic numbers, and scientific evidence.^52^ The scenarios were created with the same baseline models to analyze the disease spread of each of the pathogens of interest. In order to answer the research question, “What is the role of air travel in disease transmission as a vector for humans while carrying an infectious disease?” disease spread was modeled with and without air travel while maintaining ground transportation. In this project, individuals arrived at New York's John F. Kennedy International Airport, the fifth busiest airport in the United States and a common entry point from many international air routes. The number of passengers was varied and modeled to determine the effects of the number of initial cases. Two initial case scenarios were modeled: first, the index case to determine the effect when only 1 passenger is infected, and, second, 10 initial cases, demonstrating passengers with possible in-flight disease transmission or multiple passengers arriving from an outbreak area.

In addition, hypothetical natural pneumonic plague outbreaks were modeled starting with 1 or 10 initial cases as well as bioterrorist attacks with *Y. pestis* that may infect 1, 10, 50, 100, or 1,000 individuals in the New York area to determine the potential spread over a 6-month period.^52^ The baseline scenarios were created with the same parameters to analyze the natural progression of each of the 4 pathogens. Though real life mirrors stochastic models that allow for random changes, to understand the impact of diseases in the context of this project, the factors of season and population remained constant. The control of the models allowed the analysis of the differences between the diseases instead of the differences between the country populations or the impact of the environment.

Overall, the baseline scenarios of SARS, H1N1, Ebola, and pneumonic plague were compared against each other.^52^ All the graphical and numerical results indicated that SARS and H1N1 have a much greater impact in terms of infections and deaths than Ebola or pneumonic plague, regardless of the initial number of infections. Modeling showed that the spread of pneumonic plague is minimal and should not be a major air travel concern if an individual becomes infected. Due to the rapid progression of pneumonic plague and the high likelihood of death, spread of the disease is highly unlikely to progress from the initial victims.

## Currently Active Projects

### Parasitic Disease in Livestock in Great Britain: Evaluating Control Strategies

Endo- and ectoparasite-mediated diseases in livestock, such as gastrointestinal infection, blowfly strike, tick-borne diseases and sheep scab, cause annual losses of millions of pounds to the industry in Great Britain^60,61^ and worldwide.^62^ With resistance developing to some of the main parasiticides,^63,64^ in addition to climate change affecting the survival and seasonal dynamics of parasites,^65^ it is likely that these losses will increase in the coming years. When deciding on the best control strategies for parasitic diseases, it is often important to consider regional differences in transmission, as well as other spatial influences.

A parasitic disease model currently being built in STEM makes use of the model creator^66^ in order to capture the unique disease dynamics of parasites. The model also uses the Pajek graph creator STEM provides^67^ to build networks of farms, from a 2-farm level to a national level (in Great Britain), allowing different scenarios of treatment to be explored. The model is able to be run in batch mode with the new stochastic solver^68^ to capture the inherent variability of epidemics. In future, the impact of insecticide resistance on the efficacy of control methods will be included in the model. Results from the parasitic disease model will be able to provide insight into the control strategies that are most effective in reducing their transmission.

### African Swine Fever: Protecting Food Production in Europe

African swine fever (ASF) is an emerging infectious disease in domestic pigs (*Sus scrofa domesticus*) and wild boars (*Sus scrofa*). The morbidity and mortality rates are high in European pig herds and wild boar populations. Transmission is mainly driven by direct contact between animals or indirect contact through contaminated food and feed (discarded meat waste), infected animal residues, and humans tracking through infected areas. Following an initial outbreak in Eastern Europe in 2007, the disease spread to Poland and the Baltic countries in 2014. By 2017 ASF-infected wild boars were reported in West Ukraine, and ASF-infected swine at the Romanian-Hungarian border.^69,70^

The spread of ASF threatens agricultural economies across Europe. Germany, a country with traditionally high pig production, is at risk of experiencing significant economic losses,^71,72^ as is Hungary, where the swine sector has been a driver of recent growth of the agricultural economy. Some measures such as fencing or intense hunting of wild boars are already in place to stop the spread of ASF, but their effectiveness is questionable, and vaccines have yet to be developed. Tools and strategies to protect animal production are critically needed.

Scientists at the German Federal Institute for Risk Assessment (BfR) and Hungary's National Food Chain Safety Office explored whether STEM could be used to model the ASF epidemic and evaluate potential interventions. Their efforts built on work done by Barongo and colleagues in their study of ASF in free ranging African pig herds.^73^

As proof of concept, the project team at BfR re-implemented and adapted Barongo's basic and bio-intervention scenarios using STEM's model creator feature^66^ and ran 1,000 simulations using STEM's batch experiments feature.^68^ Their results for the base ASF model were consistent with Barongo's; those for the bio-intervention ASF models were not.^74^

Further work expanded the basic ASF model with the STEM graph generator^75,76^ to simulate certain spatial characteristics of wild boar populations, including migration and mixing rates for different population subgroups. These research findings suggest that STEM can be used to simulate and evaluate intervention scenarios, such as targeted hunting or fencing of wild boars in outbreak areas. While additional work based on real outbreaks and population data is required,^74^ the BfR project demonstrates that STEM can be used to re-implement published models and to extend them to include new components of interest.

### African Swine Fever: Informing Policy Decisions in Hungary

As part of the strategy established in 2013,^77^ the National Food Chain Safety Office works with a formally constituted ASF action group made up of stakeholders and experts (hunters, veterinarians, analysts) responsible for expert advice on controlling the spread of ASF in the country. In a number of risk evaluation exercises and in the preparation of a working paper for decision makers, they modified Barongo's model^73,78^ and ran STEM using geospatial data and estimates of wild boar density by area imported from the National Game Management Database at Szent István University.^79^ Based on the results from these simulations, the group's report identified areas at high and medium risk and critical control points, and they proposed enforcement actions to fight the spread of ASF for evaluation by policymakers.^80^

In 2018, 14 cases of ASF in wild boars reported in 1 internal region of the country (Heves county) were attributed to contaminated meat or food waste brought in by non-EU workers. Twelve new cases were identified in Szabolcs-Szatmár-Bereg county; the first case was found within 1 kilometer of the Ukrainian common border, suggesting natural spread (migration of wild boars) was the most likely source of infection.^80^ Today, the National Food Chain Safety Office is continuing its efforts.

## Conclusion and Outlook: The Challenges of Complexity

Today, STEM is one of more than 350 open source projects for which the Eclipse Foundation is home.^81^ As such, STEM benefits from Eclipse infrastructure, intellectual property management, and development community support services that protect the integrity of the STEM framework and its users.

STEM 4.0.0, released in early 2019, is supported by technical advances in the Equinox framework.^4^ Underlying Eclipse technologies that bring complexity to the code base are compensated by Eclipse tools that automatically generate the necessary source code to create new model components/modules. Specifically, the graphical model creator/ generator^6,66^ provides automatic code generation and hides much of the complexity that is inevitable in any software framework when users need to extend the available predefined model templates by adding, for example, compartments (nodes), connections (edges), or equations.

Yet, the sheer number of predefined model components, the growing number of ready-to-use models, and the increasing number of software features bring a high level of complexity and the need for extensive documentation. A user new to STEM may find the array of options difficult to navigate and be challenged to determine whether a desirable feature exists and is available to use/re-use in modeling. For new and seasoned users alike, the STEM community provides web-based forums and tools to ask questions and get pointers.

The user can benefit from a practically validated, free, open software framework capable of performing complex spatial analysis with high-resolution data as well as model-based simulations with multiple populations (and even multiple diseases) and/or large amounts of denominator data. In addition, STEM provides full transparency, reproducibility, and re-usability of models as well as complete documentation and annotation of simulation results. In the end, the STEM community promotes information exchange and knowledge integration and accelerates research in the epidemiologic modeling domain into the future.

## References

[1] The Spatiotemporal Epidemiological Modeler (STEM) Project. www.eclipse.org/stem Accessed 718, 2019

[2] Jenness SM, Goodreau SM, Morris M EpiModel: an R package for mathematical modeling of infectious disease over networks. J Stat Softw 2018;84(8):1-47. http://idmod.org/software Accessed 718, 2019 29731699 10.18637/jss.v084.i08 PMC5931789

[3] Bershteyn A, Klein DJ, Wenger E, Eckhoff PA Description of the EMOD-HIV model v0.7. arXiv:1206.3720. Accessed 718, 2019

[4] Equinox OSGi. http://www.eclipse.org/equinox/ Accessed 718, 2019

[5] Eclipse Public License 2.0. http://www.eclipse.org/legal/epl-2.0 Accessed 718, 2019

[6] The STEM Model Generator (video). https://www.youtube.com/watch?v=MtQlS7g7Qnw Accessed 718, 2019

[7] Downloadable Scenarios. https://www.eclipse.org/stem/downloads.php#DownloadableScenarios Accessed 718, 2019

[8] STEM Data. https://wiki.eclipse.org/Welcome_STEM_Developers#STEM_Data Accessed 718, 2019

[9] Dushoff J, Plotkin JB, Levin SA, Eam JD Dynamic resonance can account for seasonality of influenza epidemics. Proc Natl Acad Sci U S A 2004;101(48):16915-16916 15557003 10.1073/pnas.0407293101 PMC534740

[10] Truscott J, Fraser C, Hinsley W, et al. Quantifying the transmissibility of human influenza and its seasonal variation in temperate regions. PLoS Curr 2009;1:RRN1125 20029663 10.1371/currents.RRN1125 PMC2771768

[11] Ortega JAA, Velazquez JGM, Renly SR, Edlund SB, Kaufman JH Improving disease surveillance capabilities through a public health information affinity domain. In: Proceedings of the 1st ACM International Health Informatics Symposium ACM; 2010:536-540

[12] Edlund S, Bromberg M, Chodick G, et al. A spatiotemporal model for influenza. In: HIC 2009, Frontiers of Health Informatics. Canberra, Australia; August 19-21, 2009

[13] Edlund S, Kaufman J, Lessler J, et al. Comparing three basic models for influenza. Epidemics 2011;3(3-4):135-142 22094336 10.1016/j.epidem.2011.04.002

[14] Running an Automated Experiment. https://wiki.eclipse.org/Running_an_Automated_Experiment Accessed 718, 2019

[15] UsaMexicoDemo (file). https://www.eclipse.org/stem/download_sample.php?file=UsaMexicoDemo.zip Accessed 718, 2019

[16] Cash JR, Karp AH A variable order Runge-Kutta method for initial value problems with rapidly varying right-hand sides. ACM Trans Math Softw 1990;16:201-222

[17] Cauchemez S, Valleron AJ, Boëlle PY, Flahault A, Ferguson NM Estimating the impact of school closure on influenza transmission from sentinel data. Nature 2008:452(7188):750-754 18401408 10.1038/nature06732

[18] British Broadcasting Corporation. Measles outbreak in maps and graphics. May 2, 2013. https://www.bbc.com/news/health-22277186 Accessed 718, 2019

[19] Edlund S, Lovett D, Kaufman J, Yagci Sokat K, van Wijgerden J, Poots AJ Supporting decision making: modeling and forecasting measles in a London borough. bioRxiv 2018. doi: 10.1101/497800

[20] Measles Transmission Model. https://wiki.eclipse.org/Measles_Transmission_Model Accessed 718, 2019

[21] Mossong J, Hens N, Jit M, et al. Social contacts and mixing patterns relevant to the spread of infectious diseases. PLoS Med 2008;5(3):e74 18366252 10.1371/journal.pmed.0050074 PMC2270306

[22] Sample Configuration: H7N9 Zip. https://www.eclipse.org/stem/download_sample.php?file=H7N9.zip Accessed 718, 2019

[23] Martcheva M. Avian flu: modeling and implications for control. J Biol Sys 2014;22:151-175. doi:10.1142/S0218339014500090

[24] H7N9 Models (file). https://www.eclipse.org/stem/download_sample.php?file=H7N9.zip Accessed 718, 2019

[25] H7N9 Scenarios (file). https://www.eclipse.org/stem/download_sample.php?file=H7N9.zip Accessed 718, 2019

[26] Centers for Disease Control and Prevention. Dengue. Last reviewed May 3, 2019. http://www.cdc.gov/dengue/ Accessed 718, 2019

[27] Recker M, Blyuss KB, Simmons CP, et al. Immunological serotype interactions and their effect on the epidemiological pattern of dengue. Proc Biol Sci 2009;276(1667):2541-2548 19369266 10.1098/rspb.2009.0331 PMC2684681

[28] World Health Organization. Dengue and severe dengue. April 15, 2019. http://www.who.int/mediacentre/factsheets/fs117/en/#S Accessed 718, 2019

[29] Kawaguchi I, Sasaki A, Boots M Why are dengue virus serotypes so distantly related? Enhancement and limiting serotype similarity between dengue virus strains. Proc Biol Sci 2003;270(1530):2241-2247 14613610 10.1098/rspb.2003.2440 PMC1691498

[30] Dengue Scenarios. https://www.eclipse.org/stem/download_sample.php?file=DengueScenarios.zip Accessed 718, 2019

[31] Hu K, Thoens C, Bianco S, et al. The effects of antibody-dependent enhancement, cross immunity, and vector population on the dynamics of dengue fever. J Theor Biol 2013;319:62-74 23206388 10.1016/j.jtbi.2012.11.021

[32] Bianco S, Shaw LB, Schwartz IB Epidemics with multistrain interactions: the interplay between cross immunity and antibody-dependent enhancement. Chaos 2009;19(4):043123 20059219 10.1063/1.3270261 PMC4108630

[33] Nisalak A, Endy TP, Nimmannitya S, et al. Serotype-specific dengue virus circulation and dengue disease in Bangkok, Thailand from 1973 to 1999. Am J Trop Med Hyg 2003;68(2):191-202 12641411

[34] Kamo M, Sasaki A The effect of cross-immunity and seasonal forcing in a multi-strain epidemic model. Physica D 2002;165(3-4):228-241. 10.1016/S0167-2789(02)00389-5 Accessed 718, 2019

[35] Community Ebola Modeling Phone Call. https://wiki.eclipse.org/Community_Ebola_Modeling_Phone_Call Accessed 718, 2019

[36] Ebola Call Participants. https://wiki.eclipse.org/Ebola_Call_Participants Accessed 718, 2019

[37] Hu K, Bianco S, Edlund S, Kaufman J The impact of human behavioral changes in the 2014 West Africa Ebola outbreak. Soc Comput Behav Cult Model Predict 2015;9021:75-84

[38] Ebola Models Single Archive. https://www.eclipse.org/stem/download_sample.php?file=EbolaModelsSingleArchive.zip Accessed 718, 2019

[39] Biek R, Walsh PD, Leroy EM, Leslie LA Recent common ancestry of Ebola Zaire virus found in a bat reservoir. PLoS Pathog 2006;2(10):e90 17069458 10.1371/journal.ppat.0020090 PMC1626099

[40] Eisenberg MC, Eisenberg JNS, D'Silva JP, et al. Forecasting and uncertainty in modeling the 2014-2015 Ebola epidemic in West Africa. arXiv 2015;arXiv:1501.05555

[41] Yang W, Zhang W, Kargbo D, et al. Transmission network of the 2014-2015 Ebola epidemic in Sierra Leone. J R Soc Interface 2015;12(10). 10.1098/rsif.2015.0536 Accessed 718, 2019 PMC4685836 26559683

[42] Nieddu GT, Billings L, Kaufman JH, Forgoston E, Bianco S Extinction pathways and outbreak vulnerability in a stochastic Ebola model. J R Soc Interface 2017;14(127):20160847 28202592 10.1098/rsif.2016.0847 PMC5332568

[43] Ebola Zoonotic Reintroduction. https://www.eclipse.org/stem/download_sample.php?file=EbolaZoonoticReIntroduction.zip Accessed 718, 2019

[44] Edlund S, Davis M, Douglas JV, et al. A global model of malaria climate sensitivity: comparing malaria response to historic climate data based on simulation and officially reported malaria incidence. Malar J 2012;11:331 22988975 10.1186/1475-2875-11-331 PMC3502441

[45] MacDonald G. The Epidemiology and Control of Malaria. London: Oxford University Press, 1959.

[46] Global Earth Science Plug-in. https://www.eclipse.org/stem/download_sample.php?file=GlobalEarthScience121.zip Accessed 718, 2019

[47] Falenski A, Filter M, Thöns C, et al. A generic open-source software framework supporting scenario simulations in bioterrorist crises. Biosecur Bioterr 2013;11(Suppl 1):134-145 10.1089/bsp.2012.0071 23971799

[48] Transformation Decorator Feature. https://wiki.eclipse.org/STEM_Food_and_Food_Borne_Disease#Transformation_Decorators Accessed 718, 2019

[49] Scenario for Modeling the Transmission of Salmonella. http://wiki.eclipse.org/Sample_Projects_available_for_Download#2._A_scenario_for_modelling_the_transmission_of_Salmonella_to_pigs.2C_pork_and_humans Accessed 718, 2019

[50] Eclipse Foundation. Air Travel Model. https://wiki.eclipse.org/Air_Travel_Model Accessed 718, 2019

[51] Lessler J, Kaufman JH, Ford DA, Douglas JV The cost of simplifying air travel when modeling disease spread. PLoS One 2009;4(2):e4403 19197382 10.1371/journal.pone.0004403 PMC2633616

[52] Sevilla NL. Germs on a Plane: The Transmission and Risks of Airplane-Borne Diseases. George Mason University: ProQuest Dissertations Publications, 2017

[53] Wilder-Smith A, Freedman DO Confronting the new challenges in travel medicine: SARS. J Travel Med 2003;10(5):257-258 14531976 10.2310/7060.2003.2669 PMC7107584

[54] Khan K, Arino J, Hu W, et al. Spread of a novel influenza A (H1N1) virus via global airline transportation. N Engl J Med 2009;361(2):212-214 19564630 10.1056/NEJMc0904559

[55] Briand S, Bertherat E, Cox P, et al. The international Ebola emergency. N Engl J Med 2014;371(13):1180-1183 25140855 10.1056/NEJMp1409858

[56] Mangili A, Gendreau MA Transmission of infectious diseases during commercial air travel. Lancet 2005;365(9463):989-996 15767002 10.1016/S0140-6736(05)71089-8 PMC7134995

[57] Kool JL. Risk of person-to-person transmission of pneumonic plague. Clin Infect Dis 2005;40(8):1166-1172 15791518 10.1086/428617

[58] Dembek ZK, ed. Medical Management of Biological Casualties Handbook. 7th ed. Fort Detrick, MD: US Army Medical Research Institute of Infectious Diseases; 2011

[59] Inglesby TV, Dennis DT, Henderson DA, et al. Plague as a biological weapon: medical and public health management. JAMA 2000;283(17):2281-2290 10807389 10.1001/jama.283.17.2281

[60] Nieuwhof GJ, Bishop SC Costs of the major endemic diseases of sheep in Great Britain and the potential benefits of reduction in disease impact. Animal Sci 2005;81:23-29

[61] Bennett R, Ijpelaar J Updated estimates of the costs associated with thirty four endemic livestock diseases in Great Britain: a note. J Agricul Econ 2005;56(1):135-144

[62] Lopes LB, Nicolino R, Capanema RO, Oliveira CSF, Haddad JPA, Eckstein G. Economic impacts of parasitic diseases in cattle. CAB Reviews 2015;10(051)

[63] Doherty E, Burgess S, Mitchell S, Wall R First evidence of resistance to macrocyclic lactones in Psoroptes ovis sheep scab mites in the UK. Vet Rec 2018;182(4):106 29317477 10.1136/vr.104657

[64] Geurden T, Chartier C, Fanke J, et al. Anthelmintic resistance to ivermectin and moxidectin in gastrointestinal nematodes of cattle in Europe. Int J Parasitol Drugs Drug Resist 2015;5(3):163-171 26448902 10.1016/j.ijpddr.2015.08.001 PMC4572401

[65] Short EE, Caminade C, Thomas BN Climate change contribution to the emergence or re-emergence of parasitic diseases. Infect Dis (Auckl) 2017;10:1178633617732296 29317829 10.1177/1178633617732296 PMC5755797

[66] STEM Model Creator. https://wiki.eclipse.org/STEM_Model_Creator Accessed 718, 2019

[67] Importing a Pajek Graph. https://wiki.eclipse.org/Importing_a_Pajek_Graph Accessed 718, 2019

[68] STEM Solvers. https://wiki.eclipse.org/STEM_Solver_Stochastic_Solvers_in_Batch_Mode Accessed 718, 2019

[69] World Organization for Animal Health (OIE). African Swine Fever, Czech Republic. Follow-up Report No. 4 (OIE Report No, 24329) 2017. http://www.oie.int/wahis_2/temp/reports/en_fup_0000024329_20170719_104420.pdf Accessed 718, 2019

[70] World Organization for Animal Health (OIE). African Swine Fever, Romania. Follow-up Report No. 1 (OIE Report No, 24464) 2017. http://www.oie.int/wahis_2/public/wahid.php/Reviewreport/Review?page_refer=MapFullEventReport&reportid=24464 Accessed 718, 2019

[71] Halasa T, Botner A, Mortenson S, Christensen H, Toft N, Boklund A Simulating the epidemiological and economic effects of an African swine flu epidemic in industrialized swine populations. Vet Microbiol 2016;193:7-13 27599924 10.1016/j.vetmic.2016.08.004

[72] German Federal Ministry of Food and Agriculture (BMEL). Questions and Answers on African Swine Fever. https://bmel.de/EN/Animals/AnimalHealth/_Texte/ASP_EN.html Accessed 19, 2019

[73] Barongo MB, Bishop RP, Fevre EM, Knobel DL, Ssematimba A A mathematical model that simulates control options for African Swine Fever Virus (ASFV). PLoS One 2016;11(7):e0158658 27391689 10.1371/journal.pone.0158658 PMC4938631

[74] Guenther T, Schafft H, Filter M. Model-based analyses of the spread of African swine fever in wild boars (Sus scrofa L.) Presentation at the European Food Safety Authority (EFSA) conference, Risk Valuation and Risk Management Tools in the Agri-food Business. Hygiena Alimentorum XXXVIII: Kosice, Slovak Republic; October 17-19, 2018

[75] African Swine Fever Model. https://www.eclipse.org/stem/download_sample.php?file=ASF_models.zip Accessed 718, 2019

[76] African Swine Fever Documentation. https://wiki.eclipse.org/African_Swine_Fever Accessed 718, 2019

[77] Ministry of Rural Development. The Hungarian Agriculture and Food Industry in Figures: 2012. http://2010-2014.kormany.hu/download/0/1d/f0000/MM_2012_angol_webre.pdf Accessed 718, 2019

[78] Probst C, Globig A, Knoll B, Conraths FJ, Depner K. The behaviour of free ranging wild boar toward their dead fellows: potential implications for the transmission of African swine fever. R Soc Open Sci 2017;4:170054. 10.1098/rsos.170054 PMC5451812 28573011

[79] The National Game Management Database (NGMD). Szent Istvan University (Hungary) 1996-. http://ova.info.hu/index-en.html Accessed 19, 2019

[80] Ministry of Agriculture European Union. Short overview of epidemiological situation regarding African swine fever in Hungary. Meeting of the Standing Committee of Plants, Animals, Food and Feed. Brussels; July 12-12, 2018 https://ec.europa.eu/food/sites/food/files/animals/docs/reg-com_ahw_20180712_pres_asf_hun.pdf Accessed 718, 2019

[81] Eclipse Foundation. https://www.eclipse.org/org/ Accessed 718, 2019

